# Effects of cavity reconstruction on morbidity and quality of life after canal wall down tympanomastoidectomy^☆^

**DOI:** 10.1016/j.bjorl.2017.07.007

**Published:** 2017-08-19

**Authors:** Sinan Uluyol, Omer Ugur, Ilker Burak Arslan, Ozlem Yagiz, Murat Gumussoy, Ibrahim Cukurova

**Affiliations:** a Van Training and Research Hospital, Department of Otorhinolaryngology, Van, Turkey; b Tepecik Training and Research Hospital, Department of Otorhinolaryngology, Izmir, Turkey

**Keywords:** Caloric test, Epithelial migration, Quality of life, Prova calórica, Migração epitelial, Qualidade de vida

## Abstract

**Introduction:**

Canal wall down tympanomastoidectomy is commonly used to treat advanced chronic otitis media or cholesteatoma. The advantages of canal wall down mastoidectomy are excellent exposure for disease eradication and postoperative control of residual disease; its disadvantages include the accumulation of debris requiring life-long otological maintenance and cleaning, continuous ear drainage, fungal cavity infections, and the occurrence of dizziness and vertigo by changing temperature or pressure.

**Objective:**

To evaluate whether cavity-induced problems can be eliminated and patient comfort can be increased with mastoid cavity reconstruction.

**Methods:**

In total, 11 patients who underwent mastoid cavity reconstruction between March 2013 and June 2013 comprised the study group, and 11 patients who had dry, epithelialized CWD cavities were recruited as the control group. The study examined three parameters: epithelial migration, air caloric testing, and the Glasgow Benefit Inventory. Epithelial migration, air caloric testing, and the Glasgow Benefit Inventory were evaluated in the study and control groups.

**Results:**

The epithelial migration rate was significantly faster in study group (1.63 ± 0.5 mm/week) than control group (0.94 ± 0.37 mm/week) (*p* = 0.003, *p* < 0.05). The mean slow component velocity of nystagmus of the study group (13.33 ± 5.36°/s) was significantly lower when compared to control group (32.11 ± 9.12°/s) (*p* = 0.018). The overall the Glasgow Benefit Inventory score was −7.21, and the general subscale, physical and social health scores were −9.71, −21.09, and +20.35, respectively in the control group. These were +33.93, +35.59, +33.31, and +29.61, respectively in the study group. All but the social health score improved significantly (0.007, 0.008, 0.018, and 0.181, respectively).

**Conclusions:**

Cavity reconstruction improves epithelial migration, normalizes caloric responses and increases the quality of life. Thus, cavity rehabilitation eliminates open-cavity-induced problems by restoring the functional anatomy of the ear.

## Resumo

**Introdução:**

A timpanomastoidectomia com a técnica *Canal Wall Down*, ou técnica aberta, é comumente utilizada para tratar otite média crônica avançada ou colesteatoma. As vantagens da mastoidectomia pela técnica aberta são uma excelente exposição para a erradicação da doença e controle pós-operatório da doença residual; suas desvantagens incluem o acúmulo de detritos que requerem manutenção e limpeza otológica ao longo da vida, drenagem contínua da orelha, infecções fúngicas na cavidade e a ocorrência de tonturas e vertigem com alterações de temperatura ou pressão.

**Objetivo:**

Avaliar se os problemas induzidos pela cavidade podem ser eliminados e o conforto do paciente aumentado com a reconstrução da cavidade mastoide.

**Método:**

No total, 11 pacientes submetidos à reconstrução da cavidade mastoide entre março de 2013 e junho de 2013 constituíram o grupo de estudo, e 11 pacientes com cavidades secas e epitelizadas, operadas pela técnica aberta, foram recrutados como grupo controle. O estudo analisou três parâmetros: migração epitelial, prova calórica com estimulação a ar e o questionário *Glasgow Benefit Inventory*. A migração epitelial, a prova calórica e o *Glasgow Benefit Inventory* foram avaliados nos grupos de estudo e controle.

**Resultados:**

A taxa de migração epitelial foi significativamente mais rápida no grupo de estudo (1,63 ± 0,5 mm/semana) do que no grupo controle (0,94 ± 0,37 mm/semana) (*p* = 0,003, *p* < 0,05). A velocidade média do componente lento do nistagmo no grupo de estudo (13,33 ± 5,36°/s) foi significativamente menor se comparada ao grupo controle (32,11 ± 9,12°/s) (*p* = 0,018). O escore global do *Glasgow Benefit Inventory* foi de -7,21 e os escores da subescala geral, saúde física e social foram -9,71, -21,09 e +20,35, respectivamente, no grupo controle. Esses escores foram +33.93, +35.59, +33.31 e +29.61, respectivamente, no grupo de estudo. Todos, exceto o escore de saúde social, melhoraram significativamente (0,007, 0,008, 0,018 e 0,181, respectivamente).

**Conclusões:**

A reconstrução da cavidade melhora a migração epitelial, normaliza as respostas da prova calórica e aumenta a qualidade de vida. Assim, a reabilitação da cavidade elimina os problemas induzidos por cavidades abertas ao restaurar a anatomia funcional da orelha.

## Introduction

Canal wall down (CWD) tympanomastoidectomy is commonly used to treat advanced chronic otitis media or cholesteatomas. The advantages of CWD mastoidectomy include excellent exposure for disease eradication and postoperative control of residual disease^1^, ^2^; its disadvantages include the accumulation of debris requiring life-long otological maintenance and cleaning, continuous ear drainage, cavity infections, especially with fungal pathogens, and the occurrence of dizziness and vertigo induced by changing temperature or pressure.^3^ This study examined how well cavity reconstruction surgery eliminated cavity problems and increased patient comfort by epithelial migration measurement, air caloric testing, and the Quality Of Life (QOL) assessment. To date, epithelial migration, caloric responses or QOL assessment were evaluated separately and partially in some studies which dealt with mastoid obliteration. However, all parameters have not been studied entirely and adequately in patients who undergo mastoid obliteration. Surgical reconstruction involves reconstructing the posterior wall of the external auditory canal with conchal cartilage, and partially obliterating the mastoid cavity with temporal muscle.

## Methods

This study was performed in accordance with the Helsinki Declaration of the World Medical Association and informed consent was obtained from all participants. The study was approved by the Research Ethics Committee of a tertiary referral center (n° 2013/12).

### Patients

The present study was performed between January 2013 and June 2014 in a tertiary referral center. All of the enrolled patients were selected during routine outpatient visits. All had previously undergone a CWD mastoidectomy between 2007 and 2009 due to chronic otitis media or cholesteatoma. The potential benefits of reconstruction surgery were explained to the patients who had cavity problems such as accumulation of debris constantly and vertigo induced by temperature or pressure alterations; they were offered to take part in the study. Patients with a history of radical mastoidectomy, CWD mastoidectomy due to complications of chronic otitis media or malignant diseases were excluded. As a result, 11 patients agreed to undergo reconstruction surgery and 15 patients agreed to be in the control group. In the control group, 4 patients did not continue the epithelial migration follow-up regularly, thus 11 patients have completed the study. Patients who were recruited as the control group, had dry and epithelialized cavities. Cavity reconstruction surgeries were performed between March 2013 and June 2013. The study examined three parameters: epithelial migration, air caloric testing, and the Glasgow Benefit Inventory (GBI).

### Surgical technique

All of the patients were operated by the same otologists under general anesthesia, in order to standardize the technique. After a postauricular incision, the cavity skin was carefully elevated to avoid damaging its integrity. Osseous irregularities in the cavity were smoothed. For partial obliteration, a temporalis muscle flap with an inferior pedicle was used, and for canal wall reconstruction, a graft was prepared from conchal cartilage. When the conchal cartilage was placed as the posterior canal wall, it was supported by cartilage strips in the attic region to prevent retraction of the posterior wall. After the cavity was obliterated with temporal muscle, the cavity skin was laid on the conchal cartilage. To avoid the risk of retraction of the temporal muscle, the inferior pedicle flap was sutured to the surrounding tissues. In the first two postoperative months, all of the patients who underwent cavity reconstruction surgery had dry, epithelialized external auditory canals without any complications.

### Epithelial migration

Epithelial migration was measured by applying India ink drops in the study and control groups at the 10 week follow-up. For patients who underwent cavity reconstruction, the measurement of migration started after the rehabilitated cavity had been epithelialized and healing stages finished for approximately 6 months. The India ink drops were applied at the midpoint of the graft under an otomicroscope. Over the next 10 weeks, the patients were recalled at 2 week intervals to evaluate the epithelial migration, including the shape, direction, and speed of the migration in mm/week.

### Monothermal cold air-caloric test

Air caloric testing was performed in the study (6 months after the cavity rehabilitation surgery) and control groups. Air caloric testing was also performed before the cavity reconstruction surgery in order to measure the normalization rates of caloric responses in the study group. For the measurement, approximately 5 L of air at 24 °C was blown into the ear canal for 60 s with the patient in the 60° supine position, so that the lateral semicircular canal was vertical for optimal endolymphatic stimulation. The caloric responses were measured using Nicolet Nystar^®^ software (Nicolet Instrument Inc., Madison, USA). The nystagmus slow component velocity (SCV) was recorded in degrees/second (°/s) for 120 s. According to the software installed in the test battery, the limits of normal are between 4°/s and 20°/s. Values lower than 4°/s were considered hypo-excitability and over 20°/s hyper-excitability.

### Glasgow benefit inventory

The GBI was performed in the study (6 months after the cavity rehabilitation surgery) and control groups. The inventories were mailed to patients with a prepaid self-addressed envelope, and they were asked to return them in 1 week. The GBI was first developed by Robinson et al.^4^ to measure the change in health status due to an intervention. This questionnaire consists of 18 post-intervention questions, designed to measure changes in health status as defined by social, psychological, and physical perceptions of well-being. The questionnaire was designed as an otorhinolaryngology-specific survey.

### Statistical analysis

Statistical analysis was performed using the Statistical Package for the Social Sciences for Windows software package (ver. 20.0; SPSS Inc., Chicago, IL, USA). Continuous variables were presented as means ± standard deviation and categorical variables as percentages. The qualitative values were compared by Chi square test, the significance of different quantitative values of two groups (study group-control group or pre- and post-operative) were estimated by two-tailed *t*-tests, and the Wilcoxon signed-rank test. A *p*-value ≤0.05 was taken to indicate statistical significance.

## Results

### Demographic data

The study recruited 11 patients (11 ears) in the study group (7 women, 4 men; average age 34.89 ± 10.61 [range 19–52] years) and 11 patients (11 ears) in the control group (6 women, 5 men; average age 36.18 ± 9.84 [range 26–55] years). The demographic data of both groups did not differ statistically.

### Epithelial migration

**Figure 1:**
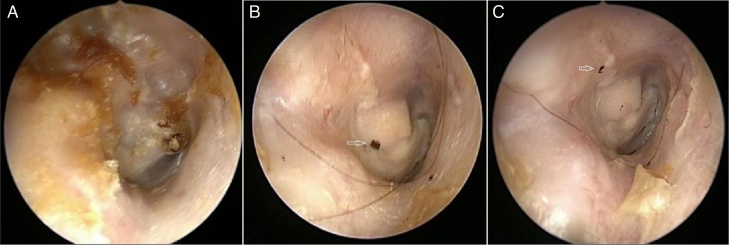
Right ear: (A) pre-operative period; (B) post-operative period; (C) epithelial migration pattern on the posterior superior direction.

In the study group, epithelial migration was observed in all canals in the posterior superior (*n* = 5, 45.4%), posterior (*n* = 4, 36.3%), and posterior inferior (*n* = 2, 18.1%) directions (Fig. 1A–C). The migration pattern was almost linear for all ears. The mean rate of migration was 1.63 ± 0.5 (range 0.7–2.56) mm/week at the end of 10 weeks follow-up. In the control group, the ink dots disappeared in three cavities at the early follow-ups and were reapplied and started to measure form the beginning; as a result, all of the patients with cavities completed the study. Migration was laterally along the facial ridge (*n* = 6, 54.5%), posterior inferiorly (*n* = 3, 27.3%), and inferiorly (*n* = 2, 18.2%). No migration was recorded in the mastoid bowls. The mean rate of migration was 0.94 ± 0.37 (range 0.4–1.57) mm/week. The migration rates significantly differed between the study group and controls (*p* = 0.003, two-tailed *t*-test) (Table 1).

**Table 1:** Migration rates and SCV values of the study and control groups.

	Study group (*n* = 11)	Control group (*n* = 11)	*p*
Migration (range)	0.7–2.56	0.4–1.57	0.003^a^
Migration rate (mm/week)	1.63 ± 0.5	0.94 ± 0.37	
SCV (range)	2–19	0–78	0.018^a^
SCV (°/s)	13.33 ± 5.362	32.11 ± 9.12	

SCV, slow component velocity.

a Two-tailed *t*-test.

### Monothermal cold air-caloric test

In the study group the mean SCV (SCV; 6 months after cavity reconstruction surgery) was significantly lower than control group (13.33 ± 5.36 vs. 32.11 ± 9.12°/s, *p* = 0.018, two-tailed *t*-test) (Table 1). The mean SCV was 27.11 ± 19.7°/s preoperatively and 13.33 ± 5.36°/s postoperatively. The difference was significant (*p* = 0.03, two-tailed *t*-test). Preoperatively, the responses were hyper-excitable in 7 (63.6%), normal in 2 (18.1%), and hypo-excitable in two patients (18.1%). After cavity rehabilitation, the response was normal in 10 (90.9%) and hypo-excitable in one patient (9.1%). Table 2 presents the normalization rates of the caloric responses.

**Table 2:** The SCV values and normalization rates of the caloric responses in pre- and post-operative periods in the study group.

	Preoperative (*n* = 11)	Postoperative (*n* = 11)	*p*
SCV (range)	2–64	2–19	0.03^a^
SCV (°/s)	27.11 ± 19.707	13.33 ± 5.362	
Normal caloric responses	2 (18.1%)	10 (90.9%)	
Abnormal caloric responses	9 (81.8%)	1 (9.1%)	

SCV, slow component velocity.

a Two-tailed *t*-test.

### Glasgow benefit inventory

In the control group, the overall GBI score was −7.21, the average general subscale score was −9.71, the physical health score was −21.09, and the social health score was +20.35. In the study group, the overall GBI score was +33.93 and the average general subscale, physical health, and social health scores were +35.59, +33.31, and +29.61, respectively. All of the differences were significant, except for the social health scores (*p* = 0.007, 0.008, 0.018, and 0.181, respectively, Wilcoxon Signed Ranks Test). Table 3 summarizes the GBI scores of the study and control groups.

**Table 3:** GBI scores of the study and control groups.

	Study group (*n* = 11)	Control group (*n* = 11)	*p*
Overall scores	+33.93	−7.21	0.007^a^
General subscale scores	+35.59	−9.71	0.008^a^
Physical health scores	+33.31	−21.09	0.018^a^
Social health scores	+29.61	+20.35	0.181^a^

GBI, Glasgow Benefit Inventory.

a Wilcoxon signed-rank test.

## Discussion

The goal of mastoid cavity obliteration is to achieve a dry, self-cleaning, functional ear canal. Many mastoid obliteration methods have been introduced to overcome cavity problems. The materials used to fill the cavity include muscle flaps, cortical bone, bone pate,^5^ cartilage,^6^ hydroxyapatite,^7^ and silicone materials.^8^ Biological materials are more resistant to infections than alloplastic materials, although they have the disadvantages of retraction, atrophy, and flap necrosis.^5^, ^9^ Gantz et al.^5^ reported that Eustachian tube dysfunction and negative pressure in the middle ear caused the retraction after mastoid obliterations. The authors supported the attic region with bone strips to prevent retraction, and suggested that the same operation be performed with cartilage strips.

For cavity reconstruction, we supported the attic and posterior mesotympanum with cartilage strips to prevent retraction due to negative pressure, and sutured the temporalis muscle pedicle to the surrounding tissue to counter the risk of muscle retraction. Our results confirmed the surgical proposals of Gantz et al.^5^ to avoid the risk of retraction. We preferred conchal cartilage for the posterior ear canal reconstruction because of its easy accessibility, low cost, and strong convex structure, which is appropriate for the ear canal.

Epithelial migration is a unique physiological self-cleaning mechanism that hinders the accumulation of debris in the ear canal. Ong et al.^10^ emphasized the rough surface and pockets in the cavity as the reason for debris accumulation on altering the epithelial migration. However, very few studies have investigated epithelial migration in open cavities. Bonding and Charabi^11^ reported a migration rate of 0.10 (range 0.02–0.45) mm/day, with migration from the center or upper part of the tympanic membrane in a lateral direction. Ong et al.^10^ reported a migration rate of 0.68 mm/week, and that the inferior lateral direction (64.7%) was the most common pattern. In both studies, there were no significant differences between open cavities and ears compared in terms of epithelial migration.

In our patients, the difference in migration rates was significant, unlike those observed in other studies. This difference might arise because the posterior superior canal wall was reconstructed in accordance with the physiology of the external ear canal; supporting the attic with cartilage avoids retraction, and the cavity skin is repositioned to form a proper ear canal. The direction of migration was mostly posterior superior in the study group versus laterally along the facial ridge in the control group; no migration was detected above the buttress level or bowl in the cavities. These results show the importance of uniformly reconstructing the posterior superior part of the ear canal for the epithelial migration and elimination of debris accumulation.

Caloric testing enables assessment of the lateral semicircular canal. Previously, water caloric tests were used, but air caloric stimulators are currently more popular. There are many reasons for using air over water. Air can be used to assess patients with eardrum perforations, external otitis, and mastoidectomy cavities, and it is technically easier to handle than water.^12^, ^13^ Air caloric testing can be monothermal or bithermal. In a detailed study of the reliability of the monothermal caloric test, Enticott et al.^14^ revealed that cold monothermal tests have >99% accuracy and emphasized that monothermal tests have the advantages of reducing test time and patient discomfort. Nishino and Granato^15^ compared air caloric testing in open cavities with normal ears, and found that the SCV with cold stimulation was 12.16°/s in normal ears versus 49.41°/s in cavities (*p* < 0.001). Beutner et al.^16^ examined patients undergoing partial mastoid obliteration pre- and post-operatively, and found that the frequency of nystagmus per minute was 72 ± 9.2 min preoperatively and 46 ± 6.2 min postoperatively. The authors suggested that obliteration of the mastoid cavity reduces caloric stimulus-induced vertigo frequency.

We obtained results similar to the studies above. In addition, we found a high rate of normalization in the caloric response when compared the pre- and post-operative values. Decreasing the semicircular canal area overlooking the ear canal and thickening the layer over the semicircular canals with cartilage and muscle likely explains this result. At the end of the study, only one patient had an abnormal caloric response. This patient had a long history of cholesteatoma, so the recurrent hypoexcitability might be due to loss of cochlear and labyrinthine function.

Traditionally, the surgical outcomes in chronic otitis media have been evaluated using the hearing results, graft survival, and eradication of disease, and only recently has the quality of life assessment begun to gain more importance. QOL assessment allows us to evaluate the success of treatments from the patient's perspective.^4^, ^17^ Very few studies have examined the QOL change with mastoid obliteration. Dornhoffer et al.^18^ studied the impact of secondary mastoid obliteration on the QOL using the GBI and three other questions about improved auditory perception, improved ear drainage, and recommending the procedure for a family member. The authors reported a total score of +28.9 (*p* < 0.001), improved auditory perception in 83%, improved ear discharge in 74%, and recommending the procedure to a family member in >90%. Kurien et al.^19^ compared primary and secondary obliteration using the GBI. The primary obliteration group had an average score of 19, general subscale score of 20, physical health score of 21, and social health score of 22. The secondary obliteration group had higher scores of 31, 34, 39, and 25, respectively. This was the first study to compare the impact of primary and secondary mastoid obliteration on the QOL.

In our study, the GBI of the control group showed the impact of the CWD tympanomastoidectomy on the QOL, whereas the GBI of the study group showed the effects of cavity reconstruction. This is the first study to make this comparison. There was a fairly good correlation between the perceived improvements in QOL. Except for the social subscale, the differences between the GBI scores of the two groups were all significant. In the study group, the most improvement was seen in the overall and physical subscale scores, reflecting the success of cavity rehabilitation at eliminating cavity-induced physical problems.

The strength of our study is the assessment of epithelial migration, caloric testing and QOL as a whole in patients who underwent cavity reconstruction for the first time. However, small sample size limits the interpretation and generalizability in the present study. Further studies with larger groups would be more beneficial to confirm our results. The main limitation was the lack of pre- and post-operative data of all parameters in cavity reconstruction group; this type of information could reflect the effect of cavity reconstruction on epithelial migration and QOL more accurately. Another limitation was the absence of long term follow-up of reconstructed cavities; it is needed to determine if the benefits of the surgical intervention are durable.

## Conclusions

In summary, the major outcomes of this study are as follows:

Reconstruction of the posterior–superior part of the ear canal seems important in terms of epithelial migration and elimination of debris accumulation. In addition, a smaller and repositioned cavity improves the epithelial migration.
Cavity reconstruction increases the tendency to normalize the abnormal caloric responses and to improve the QOL of the patients.
Cavity reconstruction seems to be a safe surgical technique that eliminates the open-cavity-induced problems by restoring the functional anatomy of the ear.

## Conflicts of interest

The authors declare no conflicts of interest.
